# CSP em 40 anos de publicação científica

**DOI:** 10.1590/0102-311XPT076324

**Published:** 2024-07-29

**Authors:** Luciana Dias de Lima, Marilia Sá Carvalho, Luciana Correia Alves

**Affiliations:** 1 Escola Nacional de Saúde Pública Sergio Arouca, Fundação Oswaldo Cruz, Rio de Janeiro, Brasil.; 2 Programa de Computação Científica, Fundação Oswaldo Cruz, Rio de Janeiro, Brasil.; 3 Instituto de Filosofia e Ciências Humanas, Universidade Estadual de Campinas, Campinas, Brasil.

**Keywords:** Publicação Periódica, Comunicação e Divulgação Científica, Saúde Pública, Periodical, Scientific Communication and Diffusion, Publica Health, Publicación Periódica, Comunicación y Divulgación Científica, Salud Pública

## Abstract

*Cadernos de Saúde Pública* (CSP) completa, em 2024, 40 anos de publicação ininterrupta. Este artigo analisa a trajetória da revista e projeta o futuro diante dos desafios contemporâneos da publicação científica do campo da Saúde Coletiva. O estudo foi desenvolvido com base na análise dos principais marcos da política editorial e na identificação dos temas mais publicados. Três períodos foram delimitados. No “início” (1985 a 1990), a revista tinha circulação restrita, com periodicidade trimestral. A palavra “epidemiologia”, usada em contexto descritivo, se sobressai. Segue-se o período de “consolidação” (1991 a outubro de 2012), quando CSP é indexado na base bibliográfica *Index Medicus*-MEDLINE e tem seu acervo *online* publicado pela *Scientific Electronic Library Online* (SciELO), ampliando o alcance dos artigos junto à academia. Nesse momento, torna-se visível o conjunto de palavras que caracterizam o campo da política, do planejamento e da gestão, e surgem os termos relacionados ao método epidemiológico. O período “diversidade” (novembro de 2012 a novembro de 2023) apresenta a obrigatoriedade de um editor externo aos quadros da Fundação Oswaldo Cruz entre os três coeditores-chefes, visando garantir a independência editorial. Novas atividades e a frequência similar entre os cinco termos mais encontrados capturam a “diversidade”. Inúmeras questões permeiam a publicação científica em 2024: Ciência Aberta não comercial, inteligência artificial, valorização da ciência, divulgação científica, entre outros. Enfrentar os novos desafios, de forma ética e transparente, permitirá avanços futuros, mantendo a credibilidade de CSP junto a autores e leitores e seu compromisso com a melhoria das condições de vida e de saúde das populações.

## Abstract (en)

In 2024, *Cadernos de Saúde Pública* (CSP) celebrates 40 years of uninterrupted publications. This article analyzes the trajectory of the journal and projects its future considering contemporary challenges in scientific publishing in the field of Public Health. This study was based on the analysis of main editorial policy milestones and the identification of the most popular topics. Three periods were recognized. In the “beginning” (1985 to 1990), the journal had restricted circulation, with a quarterly frequency. The word “epidemiology”, used in a descriptive context, is highlighted. Then, in the period of “consolidation” (1991 to October 2012), CSP was indexed in the Index Medicus-MEDLINE database and had its online collection published by the Scientific Electronic Library Online (SciELO), expanding the reach of articles. In this period, the words that characterize the field of policy, planning, and management were clearly seen, and terms related to the epidemiological method emerged. The period of “diversity” (November 2012 to November 2023) introduced the requirement of an external editor to the Oswaldo Cruz Foundation’s staff among the three co-editors-in-chief, aiming to ensure editorial independence. New activities and a similar frequency among the five most common terms highlight such “diversity”. Several issues are observed in scientific publishing in 2024: non-commercial Open Science, artificial intelligence, appreciation of science, scientific dissemination, among others. Addressing new challenges in an ethical and transparent manner will allow future advances, maintaining CSP’s credibility among authors and readers and its commitment to improvements in the living and health conditions of populations.

## Resumen

*Cadernos de Saúde Pública* (CSP) celebra 40 años de publicación continua en 2024. Este artículo analiza la trayectoria de la revista y hace proyecciones al futuro ante los retos contemporáneos de la publicación científica en el campo de la Salud Pública. El estudio se desarrolló a partir del análisis de los principales hitos de la política editorial y la identificación de los temas más publicados. Para ello, se dividieron en tres períodos. Al “comienzo” (1985 a 1990), la revista tenía una circulación restringida, con una periodicidad trimestral. En ese contexto, se destaca la palabra “epidemiología”. Después pasó al período de “consolidación” (1991 a octubre de 2012), en el que CSP se indexa en la base de datos *Index Medicus*-MEDLINE y tiene su colección en línea publicada por la *Scientific Electronic Library Online* (SciELO), lo que amplió el alcance de los artículos en la academia. En aquel momento se volvió perceptible el conjunto de palabras que caracterizan el campo de la política, la planificación y la gestión, además de aparecer términos relacionados con el método epidemiológico. En el período de “diversidad” (noviembre de 2012 a noviembre de 2023), hubo una necesidad de incluir a un editor externo al equipo de la Fundación Oswaldo Cruz entre los tres coeditores en jefe para garantizar la independencia editorial. Las nuevas actividades y la frecuencia similar entre los cinco términos principales capturan la “diversidad”. La publicación científica para el 2024 aborda numerosos temas: Ciencia Abierta no comercial, inteligencia artificial, apreciación de la ciencia, difusión científica, entre otros. Afrontar los nuevos retos, de forma ética y transparente, permitirá futuros avances manteniendo la credibilidad de CSP junto a autores y lectores, y su compromiso con la mejora de las condiciones de vida y salud de la población.

## Introdução

*Cadernos de Saúde Pública* (CSP) comemora, em 2024, 40 anos de publicação ininterrupta. Não é um feito assim tão simples. Em 2000, 174 revistas de acesso aberto desapareceram da Internet ^1^. Mesmo constando na base do sistema de identificação digital, o DOI, 27,7% (2 milhões) ^2^ dos artigos não estavam adequadamente preservados. Somente em 2023 mais de 10 mil artigos foram retratados ^3^.

A realidade de CSP quanto a isso é bem diferente. Todos os artigos, mesmo do período no qual o único repositório eram as bibliotecas, estão acessíveis *online* na página do periódico (https://cadernos.ensp.fiocruz.br/ojs/index.php/csp) e na *Scientific Electronic Library Online* (SciELO; https://www.scielo.br/j/csp/). Ao longo de toda a história da revista, apenas um artigo foi retratado.

A perenidade de CSP se deve primordialmente à sustentação financeira garantida pela Escola Nacional de Saúde Pública Sergio Arouca/Fundação Oswaldo Cruz (ENSP/Fiocruz). Além disso, o papel da SciELO ^4^, lançada em 1998, foi essencial para a garantia do acesso aberto ao conjunto de artigos publicados por CSP.

Um aspecto importante na sobrevivência de uma revista é a própria longevidade dos artigos nela publicados. Em algumas, principalmente as de clínica médica, os artigos são citados por pouco tempo, um ou dois anos. No campo da Saúde Coletiva, o tempo de citação é bem maior. Em CSP, a meia vida é de quase sete anos. Muitos dos artigos publicados na revista são referência essencial para a produção científica no campo. Um dos artigos mais citados é de 2010, apresentando a classificação de alimentos baseada na extensão e propósito do processamento industrial, no qual o termo “ultraprocessado” foi apresentado ^5^.

A credibilidade da ciência, dos cientistas e das publicações se baseia na confiança: dados coletados de forma adequada, abordagens teóricas e metodológicas aplicadas com rigor e resultados descritos honestamente ^6^. Nesses tempos de fábricas de artigos (*paper mills*), inúmeros métodos de detecção de fraudes são propostos e debatidos ^7^ ^,^ ^8^. Na corrida por mais e melhores algoritmos e programas de detecção de fraude e plágio, um elemento é chave: o conhecimento e a experiência da comunidade científica. É baseado no sistema de revisão por pares, com participação de bons revisores e editores reconhecidos pela comunidade científica do campo da Saúde Coletiva, que se alcança a fidedignidade das pesquisas publicadas em CSP. O grande número de autores e leitores que buscam a revista expressam essa credibilidade: autores porque consideram a qualidade do que é publicado e o alcance que seu artigo terá; e leitores pela confiança que CSP empresta aos artigos. É sobre essas bases que a ciência avança.

Essa credibilidade, construída ao longo do tempo, foi fundamental para garantir a longevidade da revista. Que outros aspectos dessa história foram importantes? Quais lições podemos extrair do passado para a construção de um futuro desejável? O objetivo deste artigo é analisar a trajetória de CSP, caracterizando diferentes momentos desde sua criação, e projetar perspectivas futuras perante os desafios contemporâneos da publicação científica do campo da Saúde Coletiva.

## Método

O estudo se ancorou em diferentes fontes e métodos de coleta e sistematização de informações.

A definição dos recortes temporais e qualificação dos diferentes momentos da trajetória foi realizada considerando os principais marcos da política editorial, expressos em capítulo seminal sobre o tema ^9^ e em diversos editoriais da revista. A conjugação da análise desses marcos e da composição do conselho de editores científicos permitiu a identificação de três períodos principais, assim denominados: “início” (1985 a 1990), “consolidação” (1991 a outubro de 2012) e “diversidade” (novembro de 2012 a novembro de 2023).

A análise quantitativa das publicações científicas, segundo idioma, seção e temas, foi feita com os dados importados diretamente a partir da página do SciELO, em HTML, para o site de CSP. O período analisado se inicia em 1º de março de 1985 e termina em 30 de novembro de 2023.

A informação da seção no qual o artigo foi publicado variou ao longo do tempo. Visando a comparabilidade, as seções originais foram agrupadas em nove categorias (Material Suplementar - Quadro S1; https://cadernos.ensp.fiocruz.br/static//arquivo/supl-ept076324_5810.pdf). Quando necessário, a reclassificação foi feita com base na leitura de uma amostra dos artigos. Em alguns casos, a seção original foi mantida devido à grande variedade de tipos de artigos nela incluídos. 

A análise temática foi realizada com base nas palavras extraídas dos títulos dos artigos na versão em português, por ser essa a mais completa. Na primeira etapa do algoritmo de mineração de texto, o pré-processamento, criou-se a base de palavras para análise. Foram considerados elegíveis para a análise substantivos ou locuções substantivas e adjetivos, desconsiderando outras classes de palavras que, embora muito recorrentes, não contribuem para a identificação dos temas abordados nas publicações nas quais ocorreu a extração. Assim, palavras das classes gramaticais artigo, preposição, conjunção e pronome foram suprimidas da análise. Além disso, nas locuções como “saúde coletiva” e “reforma sanitária” foi suprimido o espaço entre os termos para que a expressão fosse entendida como um único resultado, o que também ocorreu com aquelas que apresentavam algum caractere especial, como acentos, hífen e cedilha. Palavras e expressões que apresentavam alguma variação quanto a gênero gramatical e/ou número (singular ou plural) - por exemplo, mulher(es) ou brasileiro(s)/brasileira(s) - foram agrupadas em uma mesma forma não marcada gramaticalmente (Material Suplementar - Figura S1; https://cadernos.ensp.fiocruz.br/static//arquivo/supl-ept076324_5810.pdf).

Sobre essa base de palavras, a análise inicial consistiu na construção de tabelas de frequência. Os termos mais frequentes, acima da 50^a^ posição, foram avaliados quanto à verificação semântica e de sinonímia. Quando necessário, os termos foram substituídos ou padronizados de acordo com o significado. Por exemplo, expressões como desenho, *survey*, transversal, caso-controle e inquérito foram reclassificadas como método epidemiológico. E, assim, uma nova tabela foi gerada até que os termos fossem considerados adequados e representativos das temáticas do campo. Cada período foi analisado separadamente. No “início”, como o total de artigos é bem inferior aos demais (293 no total), foram consideradas palavras cuja frequência fosse maior ou igual a quatro. Para os demais períodos, nuvens foram construídas considerando as cinquenta palavras mais frequentes. A análise foi feita no software R (https://www.r-project.org/), utilizando a biblioteca de mineração de texto *tm* ^10^ (*text mining*) e de apresentação da nuvem de palavras *wordcloud* ^11^.

## Resultados

Primeiramente, apresentamos um balanço dos momentos e principais marcos da política editorial de CSP. Na sequência, aprofundamos a análise da dinâmica e evolução das publicações científicas do periódico a partir de informações selecionadas.

### Momentos e principais marcos da política editorial

Três momentos marcam a trajetória de CSP, ao longo dos seus 40 anos de existência.

O período “inicial”, de 1985 a 1990, caracteriza-se por forte preocupação com a disseminação de ideias e resultados dos estudos desenvolvidos, principalmente, por pesquisadores da ENSP. O embrião do projeto da revista remonta a um conjunto de textos mimeografados produzidos no início dos anos 1980 ^9^. Em janeiro de 1985, a estratégia de lançamento de CSP dá início a uma nova fase editorial da instituição, abrangendo diferentes programas e publicações em uma grande área de “atividades de extensão”, articulada com as necessidades de fortalecimento e interiorização do ensino, da pesquisa e dos serviços de saúde coletiva ^12^.

Mesmo de circulação ainda restrita e de periodicidade trimestral (quatro fascículos a cada ano), a revista se manteve aberta à colaboração de profissionais de quaisquer instituições, brasileiras e estrangeiras ^9^.

Nesse momento, a equipe de editoria científica era formada por uma comissão editorial da ENSP, composta por quatro pesquisadores e uma pesquisadora. Atuaram como coordenadores da Comissão e Editores-chefe da revista os pesquisadores: Frederico Simões Barbosa e Luiz Fernando Ferreira, de 1985 a 1989; Luiz Fernando Ferreira e Paulo Marchiori Buss, em 1989; e Sergio Koifman, em 1990.

O segundo período, de 1991 a outubro de 2012, marca a fase de “consolidação” de CSP. A preocupação com a indexação, tendo em vista a ampliação do alcance dos artigos publicados no cenário nacional e internacional, assume caráter central e estratégico. De suma importância foi a inclusão de CSP na base *Index Medicus*-MEDLINE em 1998, consultada internacionalmente na área da saúde. No mesmo ano, a disponibilização dos artigos na internet por meio da base SciELO, integrado aos sistemas de busca bibliográfica do MEDLINE, contribuiu para aumentar sua difusão junto à comunidade acadêmica.

Outras indexações em bases bibliográficas se conformaram no período, evidenciando um trabalho bem-sucedido envolvendo a equipe editorial de CSP e da base SciELO. Como reflete Coimbra Jr. ^13^ (p. 1820), uma verdadeira conquista em “*uma época em que os principais indexadores em ciências eram reticentes em incorporar títulos oriundos do chamado mundo em desenvolvimento*”.

Destaca-se que CSP esteve presente na trajetória da SciELO desde a sua fundação até os dias atuais, mantendo-se como um dos periódicos mais importantes da coleção e com grande número de acessos (cerca de 50 milhões até dezembro de 2023). A biblioteca inovou e se tornou referência importante nos debates sobre Ciência Aberta, tendo contribuído para tornar a boa qualidade das publicações científicas do Brasil reconhecida no mundo todo.

Também é nesse período que a revista expande significativamente o volume de publicações, diante de um número expressivo de submissões (mais de 1.000 artigos em 2006). Em 2001, CSP passa a ser bimensal, e em 2006, mensal, tendo sido implantado, neste ano, o Sistema de Avaliação e Gerenciamento de Artigos (SAGAS) em formato eletrônico. Outro aspecto marcante são as várias reformulações e inovações do projeto editorial da revista, que expressa não só uma preocupação estética, mas principalmente a busca do aprimoramento contínuo da qualidade das publicações. Entre outras mudanças, a partir de 1996 as capas de CSP passam a veicular imagens de cunho histórico, jornalístico ou documental, visando ampliar a comunicação das formas de organização da sociedade e das condições de vida e de saúde da população ^14^.

O momento expressa o importante trabalho desenvolvido pelo pesquisador Carlos E. A. Coimbra Jr., que permaneceu à frente da editoria chefe de CSP por mais de vinte anos. A equipe de editoria científica também foi ampliada. Em 1991, era composta por cinco membros (três pesquisadores e duas pesquisadoras), e, em outubro de 2012, por 17 (oito pesquisadores e nove pesquisadoras). Nesse momento, a revista também contou com a colaboração de Luis David Castiel, de 1998 a 2002, e de Mario Vianna Vettore, de 2007 a 2012, na função de Editor-chefe.

O terceiro período, iniciado em novembro de 2012, se mantém até os dias atuais e se caracteriza pela preocupação com a “diversidade”, que se configura pela necessidade de ampliar ainda mais a representatividade das diferentes áreas/subáreas e disciplinas que conformam o campo da Saúde Coletiva nas publicações, e diversificar a atuação de CSP para além da publicação científica. A partir de dezembro de 2012, CSP passa a contar com uma seção própria para publicação de temas conjunturais de relevância para a Saúde Coletiva e políticas públicas, e todos os resumos passam a ser publicados em três idiomas, incluindo o espanhol além dos tradicionais português e inglês ^15^.

O momento é marcado pela publicação de regimento interno de CSP, em setembro de 2012. Entre outros aspectos, o regimento estabelece a conformação de um colegiado de editoria chefe que deve ser composto por três editores, sendo dois pertencentes aos quadros da Fiocruz e um externo. Tal medida reflete a importância da independência como um princípio do trabalho editorial, de modo a assegurar a seleção das publicações segundo mérito e relevância social e de forma isenta a pressões ou demandas por favorecimento de autores vinculados à instituição mantenedora ^16^. Também fica definido o mandato de editores em sete anos, renováveis por mais sete. Além disso, o regimento estabelece como objetivo primordial de CSP a divulgação da produção científica original da Saúde Coletiva (em todos os seus componentes), as atribuições e responsabilidades de editores (chefes, associados e assistentes) e da Direção da ENSP.

O período também apresenta mudanças significativas. A partir de janeiro de 2016, CSP passa a ser publicada totalmente *online*. Investimentos foram feitos na reformulação do *site* da revista, que sofreu duas alterações ao longo do período. A última ocorreu em julho de 2023 ^17^ e permitiu que todo o acervo fosse disponibilizado não só na base SciELO, mas também nos servidores da Fiocruz, além da adaptação do *layout* da revista para todo tipo de dispositivo, do *desktop* aos *tablets* e telefones celulares, facilitando o acesso e leitura dos artigos. A disponibilização de todo o acervo no *site* foi um passo fundamental para integração de CSP ao PubMed Central (PMC), repositório digital desenvolvido e mantido pelo *National Center for Biotechnology Information/National Library of Medicine* (NCBI/NLM; Centro Nacional de Informações sobre Biotecnologia/Biblioteca Nacional de Medicina), dos Estados Unidos, que reúne artigos científicos da área de biomedicina e ciências da vida.

Outra característica importante do período se refere aos esforços empreendidos para a divulgação científica, no intuito de tornar as publicações de CSP mais acessíveis ao público não acadêmico e contribuindo para a valorização da ciência e da produção da Saúde Coletiva junto à sociedade em geral e à populações específicas ^18^. Foram desenvolvidos projetos para captação de recursos e parcerias internas e externas à Fiocruz, que viabilizaram inovações nas formas e veículos de divulgação da revista. Em 2015, a revista passa a integrar o Portal de Periódicos da Fiocruz (https://periodicos.fiocruz.br/pt-br/), que reúne e integra as revistas editadas pela instituição e realiza atividades de divulgação. Além disso, desde agosto de 2018 conta com um jornalista em sua equipe, e passa a difundir suas publicações nas redes sociais.

Ao final de 2023, as atividades de divulgação científica se tornaram rotineiras e consolidadas. Três frentes principais foram estruturadas: (i) redes sociais, incluindo Facebook (cadernosdesaudepublica), X (@CadernosSP) e Instagram (@cadernossp); (ii) assessoria de imprensa, com fluxos estabelecidos com a Agência Bori, os jornais Outra Saúde e Nexo Políticas Públicas; e (iii) produção de materiais audiovisuais relacionados ao programa *Entrevista com Autores*, divulgados no canal de YouTube da ENSP (https://www.youtube.com/user/enspcci) e em plataformas de *podcasts*.

O período também é marcado pelo engajamento de CSP nos debates coletivos relacionados aos desafios da produção científica do campo da Saúde Coletiva. Nesse sentido, ressalta-se o posicionamento crítico da revista em relação aos critérios adotados para avaliação da ciência e dos pesquisadores. Em vários editoriais do período são ressaltados os efeitos negativos do produtivismo e da hipercompetitividade para a integridade e qualidade das publicações ^19^ ^,^ ^20^ ^,^ ^21^. Em 2019, CSP se articulou com outros periódicos do Fórum de Editores Científicos da Fiocruz para a proposição de modelos alternativos para se avaliar os programas de pós-graduação ^22^. Ressalta-se que o tema da integridade sempre foi uma preocupação constante da política editorial ^23^: em 2017, CSP teve sua solicitação de afiliação ao *Committee on Publication Ethics* (COPE; Comitê de Ética em Publicações) aceita, junto com as demais revistas da Fiocruz ^20^.

A preocupação com a pós-graduação também esteve presente na implantação da iniciativa voltada para a formação de estágio em editoria científica ^24^. A experiência contribuiu para a qualificação de alunos de doutorado ^25^ e resultou na incorporação de Editora Júnior ao periódico.

Outro momento, digno de nota, foi a iniciativa do *fast-track*, em 2020, com o objetivo de agilizar a publicação de artigos relacionados à pandemia da COVID-19 ^26^. Nesse ano, CSP recebeu mais de 3 mil submissões, entre as quais 643 sobre o tema. A iniciativa permitiu dar maior visibilidade à resposta da comunidade científica diante da grave crise humanitária e sanitária vivenciada em todo o mundo.

Selando o compromisso com a equidade de gênero, expresso em vários editoriais ^27^, no período “diversidade” a equipe de editoria chefe passa a ser composta por três pesquisadoras mulheres ^28^: Claudia Travassos, Marilia Sá Carvalho e Cláudia Medina Coeli atuaram juntas na editoria chefe de novembro de 2012 até setembro de 2015. Em outubro de 2015, Luciana Dias de Lima substituiu Claudia Travassos e, em janeiro de 2022, Luciana Correia Alves passou a ocupar o lugar de editora externa à Fiocruz, substituindo Cláudia Medina Coeli.

Em relação à editoria científica, o número de Editores Associados é ampliado. Em 2013, a equipe era composta por vinte membros (11 homens e nove mulheres) e, ao final de 2023, por 42 pesquisadores (18 homens e 24 mulheres). A composição expressa a pluralidade de temas, mas, sobretudo, de abordagens e métodos das publicações.

**Figura 1:**
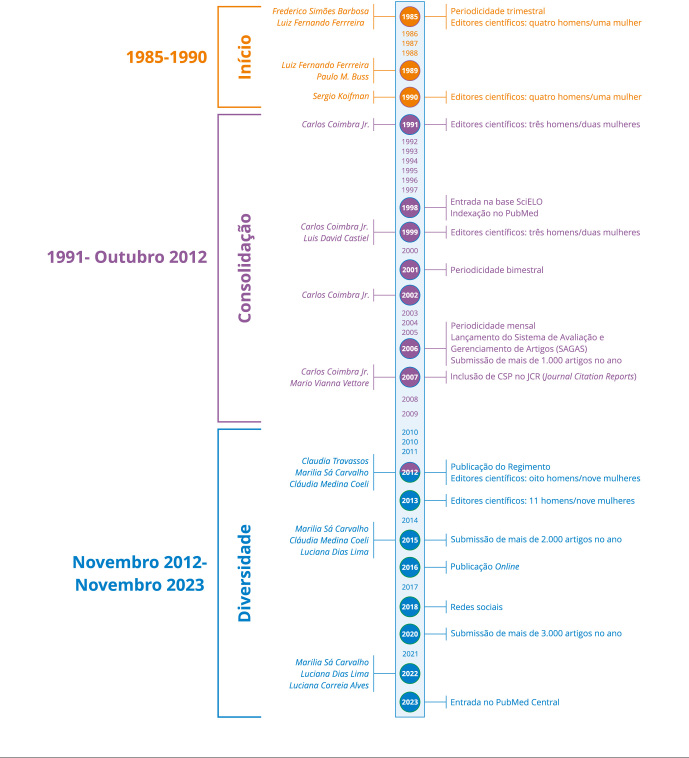
Linha do tempo de CSP, 1985 a novembro de 2023.

A Figura 1 sistematiza alguns dos marcos principais de CSP nos três períodos analisados.

### Dinâmica e evolução das publicações

Foram publicados 8.178 artigos entre o primeiro fascículo de março de 1985 até novembro de 2023. O total de manuscritos em cada período - “início”, “consolidação” e “diversidade” - foi de 293, 5.009 e 2.876, respectivamente.

**Figura 2:**
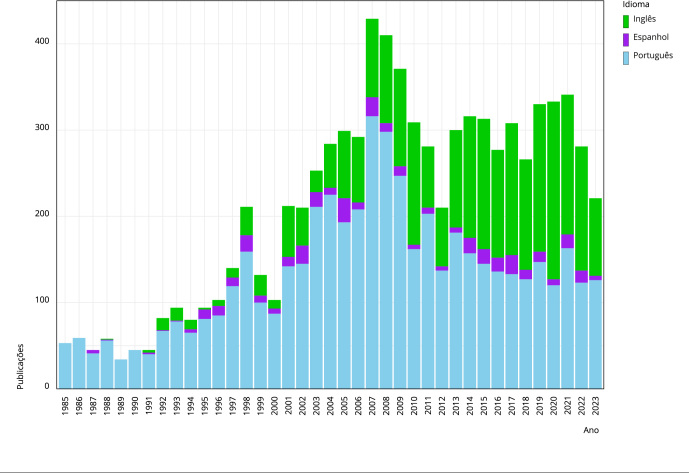
Número de publicações de CSP por ano e idioma, 1985 a novembro de 2023.

Ainda que mantida a ênfase nas publicações de manuscritos em português, observa-se uma tendência crescente de artigos publicados em inglês. O aumento se torna mais evidente a partir da segunda metade dos anos 2000, e, particularmente, na década de 2010 (Figura 2). Em relação ao espanhol, a proporção de artigos publicados nesse idioma se mantém estável, com exceções observadas em alguns anos.

Do ponto de vista de distribuição dos artigos entre as seções os períodos “consolidação” e “diversidade” apresentam perfil semelhante (Tabela 1). A quantidade de artigos classificados como “Documentos Variados” passou de 11,3% na fase inicial para cerca de 2% nos períodos posteriores. A seção “Análise e Educação” repete o mesmo padrão, inicialmente com 9,2% dos artigos período a praticamente nenhum artigo nos períodos subsequentes. Essa mesma seção inclui dois artigos classificados como seção Educação, que não deveria ser seção, uma vez que o termo indica simplesmente o conteúdo dos manuscritos.

**Tabela 1:** Artigos publicados por seções de CSP segundo período, 1985 a novembro de 2023.

Seções	Início		Consolidação		Diversidade	
	n	%	n	%	n	%
Análise e Educação	27	9,2	34	0,7	0	0,0
Artigo	138	47,1	3.550	70,9	2.008	69,8
Documentos variados	33	11,3	103	2,1	64	2,2
Editorial	20	6,8	181	3,6	135	4,7
Ensaio	0	0,0	11	0,2	73	2,5
Indução	11	3,8	467	9,3	229	8,0
Opinião e Perspectivas	39	13,3	121	2,4	128	4,5
Resenha	25	8,5	320	6,4	148	5,1
Revisão	0	0,0	222	4,4	91	3,2
Total	293	100,0	5.009	100,0	2.876	100,0

Outro aspecto interessante diz respeito à diversidade de seções do periódico. Mesmo com nomenclaturas distintas, chama atenção a abertura da revista para seções de artigos do tipo opinião, ensaios, notas, comunicações breves e perspectivas (Tabela 1).

**Figura 3:**
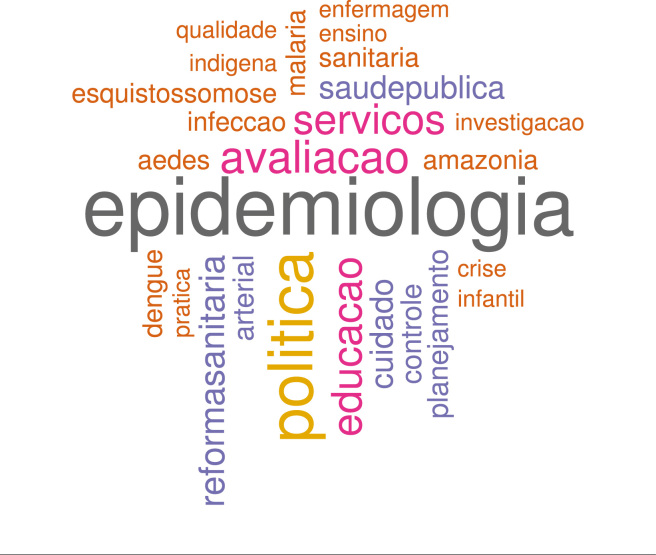
Nuvem de palavras do período “início” de CSP, 1985 a 1990.

Em cada período - “início”, “consolidação” e “diversidade” - foram analisadas, respectivamente 1.114, 30.875, 19.489 palavras. A nuvem de palavras do período “início” foi elaborada sobre 26 palavras que aparecem mais do que quatro vezes, totalizando 176 aparições (Figura 3). A palavra *epidemiologia* representa 12,5% do total, 1,4 vezes mais frequente do que *politica*, o segundo da lista. O contexto no qual “epidemiologia” surge no título dos artigos é, em geral, a descrição de prevalência de alguma doença específica analisada. Cinco palavras características das doenças transmissíveis - *infeccao*, *malaria*, *esquistossomose*, *aedes* e *dengue* - somam 14,2% do total.

Diversos termos estão relacionados à grande área hoje classificada como Política, Planejamento e Gestão (PPG) - *politica*, *avaliacao*, *servicos*, *cuidado*, *planejamento* - respondem por 28,4% do total; *reformasanitaria*, isoladamente, representa 4,6% do total de palavras incluídas na nuvem.

**Figura 4:**
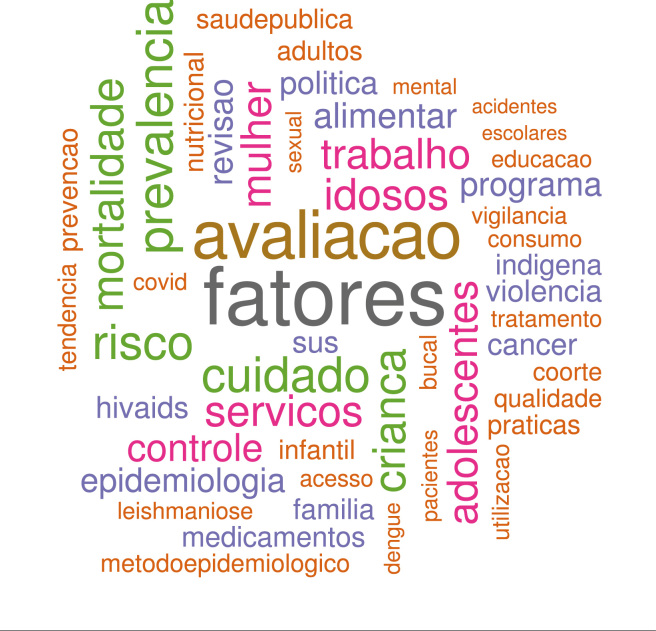
Nuvem de palavras do período “consolidação” de CSP, 1991 a novembro de 2012.

No período “consolidação”, as cinquenta palavras incluídas na nuvem tiveram frequência igual ou superior a 55 ocorrências (Figura 4). A palavra mais frequente, *fatores*, representa o conceito de “fatores de risco”, muito usual nos estudos epidemiológicos de período. Essa responde por 6,4% do total, e é 1,2 vezes mais frequente do que a palavra seguinte, *avaliacao*, que por sua vez é 1,4 vezes mais frequente que *prevalencia*. Os termos identificados na área de PPG - *cuidado*, *servicos*, *programa*, *politica*, *praticas*, *utilizacao*, *acesso* - foram responsáveis por 14,5% do total, semelhante ao período anterior.

A palavra *epidemiologia* tem frequência pequena (2,2%) e surge em contexto diferente da fase anterior, não mais relacionada à especificidade de um agravo, mas à questões conceituais tais como ecoepidemiologia ou uso da epidemiologia em circunstâncias específicas, por exemplo na avaliação. Termos em geral relacionados a estudos epidemiológicos e temas usuais nesse âmbito - *prevalencia*, *risco*, *mortalidade* - representam, somados, 10,3% do total. Termos agregados sob o rótulo “metodoepidemiologico” são característicos de desenhos de estudos epidemiológicos - caso-controle, desenho, inquérito - e representam 1,3%. A palavra *coorte*, também um desenho de estudo, foi mantida isolada das demais pela alta frequência: 1,3% do total incluído na nuvem.

Nesse período, surgem também as palavras que caracterizam populações específicas: *crianca*, *idosos*, *adolescentes*, *mulher*, *infantil*, *adultos*. Entre os agravos, perdem proeminência as doenças transmitidas por vetores e surgem, entre outras: *violencia*, *cancer*, *hivaids*, *acidentes*.

Artigos que incluem no título os termos *mental*, *bucal* e *sexual* indicam temas que aparecem nessa fase. Em 1994, a palavra *genero* compõe pela primeira vez o título de um artigo.

**Figura 5:**
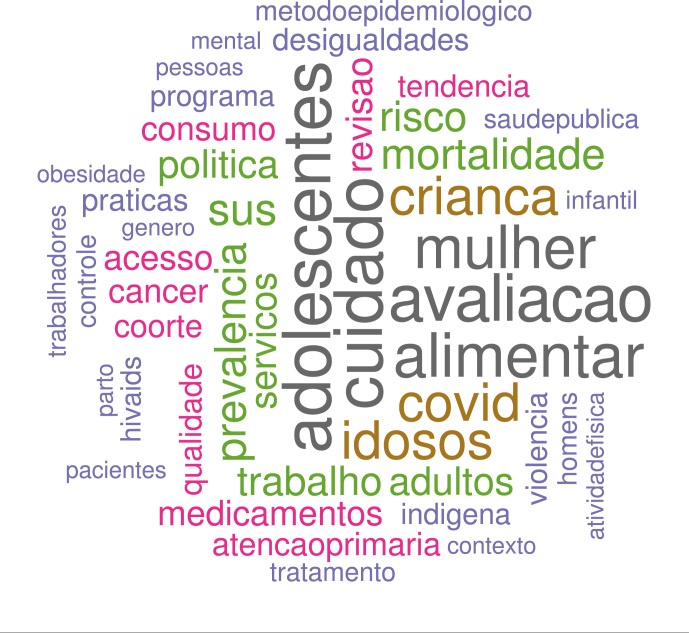
Nuvem de palavras do período “diversidade” de CSP, novembro de 2012 a novembro de 2023.

Na nuvem de palavras do período “diversidade”, entre as cinquenta palavras incluídas, compartilham posição semelhante no ranking de frequência: *cuidado*, *adolescentes*, *avaliacao*, *alimentar* e *mulher*, cada uma com cerca de 4% a 4,5% do total incluído na análise (Figura 5).

Covid, com frequência próxima das anteriores (3,4%), reflete, obviamente, a importância do tema na conjuntura, mas é também decorrente dos 62 artigos aprovados (entre os 643 submetidos) avaliados e publicados em regime de *fast-track*.

Em conjunto, além de *avaliacao* e *cuidado*, a parcela que chamamos aqui de PPG identificada pelos termos *politica*, *servicos*, *acesso*, *atencaoprimaria*, *programa*, *praticas* e *basica*, responde por 13,1% do total. O termo *sus* aparece pela primeira vez em 1989, e toma vulto nos períodos mais recentes: 1,9% e 2,7%, respectivamente.

Palavras que indicam estudos relacionados ao ciclo de vida - *criancas*, *idosos*, *adolescentes*, *mulher*, *infantil*, *adultos* - representam 16%. Ganham relevância temas relacionados aos estudos de nutrição, ficando *alimentar* com 4% do total. Tomadas em conjunto - *alimentar*, *nutricional*, *peso* e *obesidade* - essa área atinge 7,1% do total de palavras analisadas. Também nesse período surgem os termos que refletem os estudos voltados para as desigualdades na sociedade: *genero* e *desigualdades*.

Característicos de estudos epidemiológicos, os termos *prevalencia*, *risco*, *coorte*, *mortalidade* somam 9,2%; *metodosepidemiologicos* e *coorte* mantém proporção semelhante à fase anterior: 1,5% e 1,7%, respectivamente.

A frequência relativa das palavras ranqueadas em primeiro e segundo lugar em cada período aponta a diversificação que se observa no período mais recente, quando as primeiras palavras têm proporção praticamente igual. Nos períodos anteriores - “início” e “consolidação” -, a razão entre a frequência da primeira palavra em relação à segunda colocada foi de 1,4 e 1,2, respectivamente.

Estudos de saúde dos povos indígenas fizeram parte de todos os períodos analisados, sempre com frequência superior a 1,5% das palavras analisadas.

Ao longo desses 40 anos, CSP publicou 70 fascículos temáticos que possibilitam um espaço maior para conteúdos específicos.

## Discussão

A análise dos marcos da política editorial, da dinâmica e da evolução das publicações da revista permitiu identificar continuidades e mudanças, características comuns e peculiaridades, em diferentes momentos de sua trajetória. Nesses 40 anos, em meio a mudanças na política editorial e no mundo da ciência e da publicação científica, CSP manteve alguns princípios. Entre eles, ressalta-se o compromisso de manter a revista aberta à publicação de artigos de autores das mais diversas instituições e países. Também esteve presente a indução, como mecanismo de estimular a publicação de temas emergentes e ainda pouco tratados na literatura do campo da Saúde Coletiva. Além disso, a interdisciplinaridade necessária à Saúde Coletiva ^29^ encontra em CSP espaço para debate e ação ^30^.

A preocupação com a ampliação do alcance e da diversidade do público leitor da revista também se expressa nos idiomas dos textos. Cada um dos três idiomas publicados em CSP - português, inglês e espanhol - tem um público leitor e um potencial de diálogo com a comunidade do campo. Cabe uma reflexão mais aprofundada sobre o papel do inglês como a língua franca da comunicação científica. Ainda que facilite o diálogo entre cientistas de diversas origens, amplia a desigualdade que favorece os países anglófonos, em especial os Estados Unidos e o Reino Unido. Assumir o inglês como o idioma obrigatório da ciência gera consequências para os pesquisadores com outro idioma nativo, seja pelos custos de tradução ou pelas recusas devido limitações na redação ^31^. Mas talvez o maior dano da exclusão da ciência não publicada em inglês seja a perda para toda a ciência, que ignora parte da produção científica de países como o Brasil ou a China ^32^ ^,^ ^33^.

Embora as seções nas quais os artigos foram inicialmente classificados tenham tido nomenclatura diversa ao longo do tempo, o período “início” mostra um processo de publicação ainda pouco maduro, refletido na falta de tipologia clara que permitisse classificar adequadamente os manuscritos. A seção “Análise”, por exemplo, reúne um conjunto de artigos díspares, entre os quais resenhas, ensaios, opinião. A padronização dos artigos nesse período, tais como a obrigatoriedade de resumo em português e inglês segundo a definição da seção, o tamanho dos artigos, a formatação das referências bibliográficas não está presente. Por outro lado, entre 1992 e 1993, quando os artigos submetidos na fase anterior já estão publicados e a normatização está sendo implantada, fica estabelecido um padrão claro e consistente. 

A amplitude de temas abordados ao longo da história de CSP reflete os grandes debates do campo travados no Brasil. O termo *reformasanitaria*, por exemplo, permeou a discussão da política de saúde antes da criação do Sistema Único de Saúde (SUS), sendo seu embrião. Artigos publicados em 1986/1987 refletem a prioridade do tema nas páginas da revista. CSP também manteve posicionamentos claros na defesa da democracia em momentos políticos críticos, e respondeu de forma oportuna às necessidades de ampliação da publicação de temas cruciais para a Saúde Coletiva. É o caso das emergências sanitárias, como o Zika vírus em 2016 e a COVID-19 entre 2020 e 2023.

Ao longo de toda a vida do periódico, é grande a presença de um conjunto de palavras que caracterizam a área de PPG e suas vertentes, bem como objetos das Ciências Sociais e Humanas. Considerando os grandes desafios colocados para a Saúde Coletiva em publicações recentes ^34^, todos foram temas de artigos em CSP: da saúde mental às doenças infecciosas; da insegurança alimentar à diabetes. Na fase inicial, CSP focava nas doenças transmitidas por vetores; na fase da consolidação, surgem os artigos voltados para detecção de fatores de risco; e na fase seguinte, a crítica a esses mesmos métodos tão frequentes na epidemiologia ^35^. A ampliação da representatividade temática ocorreu sem prejuízo da qualidade das publicações, elemento crucial que explica a credibilidade e longevidade da revista.

Nesse momento, a publicação científica vive um cenário de grandes incertezas e contradições. Ciência e acesso livre são vitais para a construção do conhecimento. Mas, perante milhares de artigos publicados em *preprints*, a quem cabe o controle de qualidade ^36^? Mesmo considerando suas limitações, a revisão por pares ainda é o paradigma da credibilidade das publicações. No entanto, obter bons pareceres é cada vez mais difícil, como relatado por diversas revistas e editorial de CSP ^37^. Como lidar com a produção fraudulenta de artigos científicos produzidos em série? Qual será o impacto dos algoritmos de inteligência artificial generativa na redação de artigos científicos, e o que esperar do futuro? Como recuperar e aumentar a credibilidade da ciência e de cientistas diante dos ataques negacionistas recentes?

O conhecimento científico contemporâneo exige que os processos pelos quais a ciência é comunicada estejam em constante evolução e modernização. Formatos de divulgação mais dinâmicos - redes sociais, vídeos, *podcasts* e parcerias com a imprensa -, que já fazem parte do cotidiano da revista, são formas de aproximação entre a comunicação e a divulgação científicas. A diversificação do público informado pela ciência publicada nas revistas é uma das formas de aproximar cientistas da população em geral. Além disso, ampliar a inclusão e a acessibilidade, com versão em libras para os vídeos e conversão de texto para áudio, é um caminho a ser avaliado.

CSP sempre esteve comprometido com as práticas de Ciência Aberta, como um bem público não comercial ^38^. Não cobra taxa de submissão e publicação, além todo o acervo estar integralmente disponibilizado no seu *site* e na base SciELO, o denominado acesso aberto diamante. Os *preprints* anteriores à submissão passaram a ser aceitos a partir de 2020, e está em processo de implantação a abertura dos nomes dos Editores Associados responsáveis por acompanhar o processo de avaliação dos artigos. Adotar o conjunto de preceitos da Ciência Aberta, incluindo a abertura da autoria, da avaliação dos manuscritos e o compartilhamento de dados, depende da compreensão da comunidade científica do campo. Por isso, CSP realizará um inquérito sobre o tema junto aos autores e revisores que contribuem com a revista.

Entretanto, a crise de confiança e as retratações de artigos cada vez mais frequentes colocam a urgência da discussão sobre reprodutibilidade. Em primeiro lugar, ela deve garantir que, com o mesmo conjunto de dados, seja alcançado um resultado igual ao obtido pelos autores. Mas também implica na capacidade de reproduzir um estudo em outras regiões e populações ^39^. Para tal, os autores deverão informar maiores detalhes sobre os desenhos de estudo e as metodologias utilizadas, além dos códigos e os dados brutos.

Novas tendências, como o uso de ferramentas de inteligência artificial (IA) e *large language models* (LLM; grandes modelos de linguagem) como o ChatGPT, certamente serão cada vez mais utilizadas, tornando urgente os posicionamentos das revistas, de forma a adotar o uso responsável dessas tecnologias no curto e médio prazos ^40^. Os preceitos de integridade em pesquisa recomendados pelo COPE recomendam que os autores que usarem IA mencionem a etapa em que foi realizado e atribuam os créditos em seções de agradecimentos, métodos ou outros. Não cabe citá-los como coautores, pois não se pode atribuir a essas ferramentas a responsabilidade pelo que foi escrito ^41^. Ferramentas como ChatGPT e similares podem facilitar práticas que infringem a ética em pesquisa, podendo disseminar informações enganosas ou erradas, o que adiciona uma preocupação em áreas como a da Saúde Coletiva. Ainda, a aplicação dessas ferramentas pode provocar um problema adicional para os editores se não for informada pelos autores. Alguns aplicativos estão sendo lançados a fim de identificar textos gerados por esses modelos, mas ainda não se sabe sobre sua confiabilidade ^8^.

Existem várias atividades no processo editorial que podem se beneficiar com essas ferramentas. Considerando que o uso de IA não é diferente de *sites* de pesquisas na internet, de tradução e de correção de gramática e estilo textual, os autores podem usá-las para produzir textos em outro idioma, revisar os textos de acordo com a norma culta ou organizar as referências bibliográficas. Autores também poderão utilizar essas ferramentas como auxiliares em suas análises estatísticas. Por sua vez, os editores poderão lançar mão delas para detectar plágio, conflitos de interesse, localizar revisores especialistas no tema ou orientar os autores a utilizar IA para melhorar a linguagem e o estilo de um manuscrito. A IA também pode ser aplicada na fase de formatação do manuscrito. Reconhecendo que as questões éticas do uso da IA são complexas, é necessário amplo debate no sentido do estabelecimento de normas sobre o uso apropriado dessas ferramentas ^42^ ^,^ ^43^.

Esse artigo buscou analisar a trajetória e projetar perspectivas futuras para CSP. A análise de quatro décadas de publicação científica permite afirmar que, independentemente da época, os fundamentos da revista sempre foram a qualidade e a inovação. Ao mesmo tempo em que mantém esses princípios, bases da credibilidade do periódico, a revista se adaptou, respondendo às necessidades de seu tempo e da Saúde Coletiva. Inúmeras questões estão colocadas em 2024, envolvendo: Ciência Aberta não comercial, inteligência artificial, integridade em pesquisa, valorização da ciência, divulgação científica, entre outros. Enfrentar os novos desafios de forma ética e transparente, como foi feito ao longo da sua história, permitirá avanços futuros, mantendo a credibilidade de CSP junto a autores e leitores e seu compromisso com a melhoria das condições de vida e saúde das populações.
