# Supplementary material for: CSP in 40 years of scientific publications
Source: Cad Saude Publica. 2024 Jul 29;40(6):e00076324. [Article in Portuguese] doi: 10.1590/0102-311XPT076324 (PMC11290830; doi:10.1590/0102-311XPT076324)
Supplement: Supplementary file 1 [file 1678-4464-csp-40-06-PT076324-s.pdf]

Quadro S1 Reclassificação das seções.

SEÇÃO RECLASSIFICADA	SEÇÃO ORIGINAL
Análise e Educação	Análise, Educação
Artigo	Artigo, Artigo Especial, Comunicações, Comunicações Breves, Método, Notas, Pesquisa, Questões Metodológicas
Documentos Variados	Carta, Comentários, Conferencia, Depoimento, Entrevista,Obituário, Registro, Relatório, Teses
Editorial	Editorial
Ensaio	Ensaio, Tema
Indução	Debate, Espaço Temático, Fórum
Opinião e Perspectivas	Informe, Perspectivas, Opinião
Resenha	Resenha
Revisão	Revisão

Figura S1 Script: pré-processamento.

Bibliotecas

```
library(slam)
library(tm)
library(wordcloud)
library(tidyverse)
library(lubridate)
library(wordcloud)
library(slam)
library(tm)
library(stringr)
```

Pré-processamento

Exclusões

```
unico <-unico[unico$secao != "Retracao" & unico$secao != "Errata" & unico$secao != "Agradecimentos" ,  ]
```

Palavras compostas

```
unico$titulo <- str_replace(unico$titulo, “epidemiologico” , “epidemiologia”)
unico$titulo <- str_replace(unico$titulo, “epidemiologica” , “epidemiologia”)
unico$titulo <- str_replace(unico$titulo, “saude coletiva” , “SaudeColetiva”)
unico$titulo <- str_replace(unico$titulo, “reforma sanitaria” , “ReformaSanitaria”)
unico$titulo <- str_replace(unico$titulo, “saude publica”, “saudepublica”)
unico$titulo <- str_replace(unico$titulo, “politica publica”, “politicapublica”)
unico$titulo <- str_replace(unico$titulo, “recursos humanos” , “RecursosHumanos”)
unico$titulo <- str_replace(unico$titulo, “vigilancia epidemiologica”, “vigilanciaepidemiologica”)
unico$titulo <- str_replace(unico$titulo, “atencao primaria”, “AtencaoPrimaria”)
unico$titulo <- str_replace(unico$titulo, “baixo peso”, “baixopeso”)
unico$titulo <- str_replace(unico$titulo, “indice de massa corporal”, “IMC”)
unico$titulo <- str_replace(unico$titulo, “mulheres”, “mulher”)
unico$titulo <- str_replace(unico$titulo, “criancas”, “crianca”)
unico$titulo <- str_replace(unico$titulo, “politicas”, “politica”)
unico$titulo <- str_replace(unico$titulo, “amerindios”, “indigena”)
unico$titulo <- str_replace(unico$titulo, “indio”, “indigena”)
unico$titulo <- str_replace(unico$titulo, “indigenas”, “indigena”)
## Palavras excluídas “”unico$titulo <- str_replace(unico$titulo, “naoindigenas”, “indigena”)
unico$titulo <- str_replace(unico$titulo, “atividade fisica”, “atividadefisica”)
unico$titulo <- str_replace(unico$titulo, “meio ambiente”, “ambiente”)
unico$titulo <- str_replace(unico$titulo, “aedes aegypti”, “aedes”)
unico$titulo <- str_replace(unico$titulo, “aedes albopictus”, “aedes”)
unico$titulo <- str_replace(unico$titulo, “alimentacao”, “alimentar”)
unico$titulo <- str_replace(unico$titulo, “alimentos”, “alimentar”)
```

Palavras avaliadas no contexto e retiradas

```
unico$titulo[str_detect (unico$titulo,“meio”)] # “por meio de”, pouco informativa
unico$titulo[str_detect (unico$titulo,“participacao social”)] # somente 4
unico$titulo[str_detect(unico$titulo, “populacao”)] # excluir, manter tipo de população (idosa, crianças, mulheres, etc)
```

Método epidemiológico (*coorte* mantida por ser muito frequente)

```
unico$titulo <- str_replace(unico$titulo, “desenho”, “metodoepidemiologico”)
unico$titulo <- str_replace(unico$titulo, “survey”, “metodoepidemiologico”)
unico$titulo <- str_replace(unico$titulo, “inquerito”, “metodoepidemiologico”)
nico$titulo <- str_replace(unico$titulo, “transversal”, “metodoepidemiologico”)
unico$titulo <- str_replace(unico$titulo, “caso-controle”, “metodoepidemiologico”)
```

Períodos

```
unico$data <- dmy(unico$data_publicacao)
unico$data[is.na(unico$data)] <- ymd(unico$data_publicacao[is.na(unico$datas)])
unico$mes <- month(unico$data)
unico$periodo <- “Início”
unico$periodo[unico$ano >= 1991] <- “Consolidação”
unico$periodo[unico$data >= “2012-11-01”] <- “Diversidade”
table(unico$periodo)
```

Palavras excluídas

abordagem
algumas
alguns
america
analise
anos
area
artigo
aspecto
aspectos
associacao
associados
atividade
atual
bahia
base
belo
brasil
brasileira
brasileiras
brasileiro
brasileiros
capitais
caracteristicas
caso
casos
cidade
colombia
condicoes
consideracoes
contribuicao
cruz
dados
debate
desafios
desenvolvimento
doenca
doencas
durante
estado
estudo
estudos
experiencia
gerais
grande
grupos
horizonte
idade
impacto
janeiro
jesus
latina
meio
minas
municipio
municipios
nacional
nao
nordeste
nordeste
notas
nova
oswaldo
pais
parana
partir
paulo
pelotas
perfil
pernambuco
perspectiva
perspectivas
pesquisa
populacao
populacional
populacoes
pressao
projeto
proposta
propostas
publica
questao
regiao
relacao
relacionados
residente
residentes
resultados
rio
salvador
santa
sao
saude
setor
sistema
sistematica
situacao
sociais
social
sul
uso
vida
